# Retraction: Effects of microRNA-136 on melanoma cell proliferation, apoptosis and epithelial mesenchymal transition by targeting PMEL through the Wnt signaling pathway

**DOI:** 10.1042/BSR-20170743_RET

**Published:** 2021-04-23

**Keywords:** Cell proliferation, Epithelial-mesenchymal transition, Melanoma cells, microRNA-136, PMEL, Wnt signaling pathway

This Retraction follows an Expression of Concern relating to this article previously published by Portland Press.

This article is being retracted from *Bioscience Reports* at the request of the Editor-in-Chief and the Editorial Board following receipt of a notification from a reader, alerting the Editorial Board to duplication in Figure 1 as well as duplication in the Western blots of Figure 6B, of which parts appear in multiple published articles from different author groups.

The authors have been contacted in regard to the retraction, and disagree with the Journal's decision to retract the article. The authors state that the duplications are due to errors being made by the authors during the submission of the article. Given the extent of the issues raised, the Editorial Board stands by the decision to retract the article.

